# Spectrum of Fungal Infections in a Tertiary Care Centre of North India: Pre-COVID and COVID Scenario and Implications

**DOI:** 10.7759/cureus.38616

**Published:** 2023-05-05

**Authors:** Neha Singh, Rupinder Kaur, Ariba Zaidi, Sukhpreet Aulakh, Vijay S Nijhawan

**Affiliations:** 1 Pathology, Maharishi Markandeshwar Institute of Medical Science and Research, Mullana, Ambala, IND; 2 Pathology, Christian Medical College & Brown Memorial Hospital, Ludhiana, IND; 3 Pathology, Dr Ram Manohar Lohia Institute of Medical Sciences, Lucknow, IND

**Keywords:** rhino-orbital, ophthalmic exenteration, mucormycosis, fungus, covid-19

## Abstract

Background

Rhino orbital mucormycosis is a rare and very aggressive entity. A sudden rise of this entity has been noticed with the insurgence of the COVID-19 pandemic both among immunocompromised and immuno-competent patients. This study was done to determine any possible correlation between these two deadly diseases.

Materials and Methods

This was a retrospective observational study done in the pathology department of a tertiary care center in North India over a three-year period (January 2019 - December 2021). Patient details along with relevant clinical data were retrieved from the patient’s record file. Hematoxylin and eosin-stained slides of diagnosed cases were taken from the department records.

Results

A total of 45 patients (34 males, 11 females) were included in the study, seven of which were ophthalmic exenteration specimens. The mean age of the patients was 52.68 years. Fifteen cases showed COVID-19 reverse transcription-polymerase chain reaction (RT-PCR) positivity. Histopathology revealed the presence of mucormycosis in all the cases. There were six cases showing granuloma formation and 14 cases revealed mixed fungal infection. Optic nerve involvement was seen in six cases of exenteration specimens.

Conclusions

The present study showed a sudden resurgence of secondary fungal infections, especially during the second wave of the COVID-19 pandemic. Associated co-morbid conditions and injudicious use of steroids and antibiotics have been the cause of depressed immunity leading to the infections. One must be aware of such co-infections to facilitate timely medical management to reduce morbidity and mortality.

## Introduction

Severe acute respiratory syndrome coronavirus 2 (SARS-CoV-2) declared a pandemic worldwide, has presented a formidable medical challenge to the health system since 2020. Infection with CoV-2 predominantly affects the respiratory system with variable severity ranging from a common cold to more severe diseases including pneumonia [1]. The release of the danger-associated molecular pattern (DAMP) by the virus has been implicated in the exacerbation of the immune and inflammatory response within the lungs causing diffuse alveolar damage (DAD) [2]. Besides causing severe inflammatory exudation the disease is also known to cause immunosuppression with a decrease in clusters of differentiation (CD)4 T and CD8 T cell counts which could lead to secondary bacterial and fungal co-infections, especially in patients with pre-existing medical ailments like diabetes mellitus, hypertension, heart ailment, etc. [3, 4]. Of the various co-infections seen during the SARS-CoV-2 pandemic, an upsurge of fungal infections, especially by mucormycosis species involving rhino-orbital areas caused a lot of concern [5-11]. Infection in the sino-orbital areas not only caused local damage but also lead to angio-invasion, widespread necrosis, and destruction of the bone and soft tissue of the oral cavity and face. Studies showing cerebral and orbital involvement were also noted among immunocompromised individuals [1, 4, 5, 7, 9, 12-14]. Awareness regarding this fatal phenomenon is mandatory for early detection and therapeutic intervention so as to reduce morbidity and mortality [15-17]. The present study was done to delineate the clinical and epidemiological profile as well as associated risk factors of histopathologically diagnosed cases of mucormycosis contributing to this illness. The study also depicts a marked upsurge in histopathologically diagnosed fungal infection cases during the COV-2 pandemic (first and second wave) as compared to pre COVID era.

## Materials and methods

This was a retrospective observational study done in the Department of Histopathology in a tertiary care center in North India over a three-year period (January 2019- December 2021). A total of 45 proven cases of mucormycosis in both rhino-nasal biopsies, as well as ophthalmic exenteration specimens subjected to histopathological examination, were included in the study. This did not include the six cases seen in the pre-COVID era. Demographic and clinical detail documented in the patient (45 cases) record files along with detail of grossing of the specimen from the departmental records were noted as per the protocol pro-forma.

Routine laboratory investigations like complete blood count, random blood sugar, renal function test, D-dimer, and glycated hemoglobin (HBA1c) wherever available were noted down. Magnetic resonance imaging of the orbit and brain did in most of the ophthalmic exenteration specimens to assess the extent of the disease and the findings were retrieved from the patient's record file.

Smaller biopsies were measured and processed as per the laboratory protocol. Each of the ophthalmic exenteration specimens was measured and examined for any areas of necrosis or hemorrhage in the peri-orbital soft tissue. The optic nerve was identified and the resection margin was sampled and processed separately in all the cases. An anteroposterior sagittal section through the specimen was made with the identification of internal structures. Any abnormality if present was noted. The sections taken were processed as per the laboratory protocol.

Hematoxylin and eosin (H&E) stained slides along with blocks and special stain slides like Grocott-Gomori methenamine silver (GMS), and if available, periodic acid Schiff (PAS) of all the cases were retrieved from the histopathology records and reviewed to see pathological changes caused by fungus. The diagnosis of mucormycosis in sino-orbital specimens was based on the demonstration of broad septate hyphae with right angle branching on histopathological and/or 20% potassium hydroxide (KOH) preparation as well as Sabouraud's dextrose agar culture reports [18]. In case of discrepancies, fresh sections were cut from the available blocks. Special stains if required were done. The cases were reviewed by a minimum of two histopathologists from our institute. The histopathological reports were correlated with microbiological diagnosis (KOH mount and fungal culture) for confirmation of fungal type wherever possible.

Statistical analysis

This was an observational descriptive study with the use of counts and percentages. The data was plotted on the MS Excel sheet with all the analyses performed on SPSS (Statistics for Windows, Version 10 Chicago, SPSS Inc.).

## Results

Clinicopathological evaluation of a total of 45 cases (excluding pre-COVID era cases) of sino-orbital fungal infections reported on tissue biopsies (38 cases) and ophthalmic exenteration (seven cases) was done during the study period. Ophthalmic exenteration cases were encountered only during the second wave of COVID-19. A rise in these infections was seen in the present study with only six cases (13.3%) reported in the year 2019 (pre-COVID era) which rose to 14 (31.1%) during the first wave of the COVID-19 pandemic (2020) and to 31 (68.8%) in the second wave (2021).

Demographic profile

The majority of the patients in our study were males (34 cases) with male to female ratio of 3:1. The age group of the patients ranged from 23-70 years (mean age 52.68 years). Most of the patients (83.7%) presented solely with nasal cavity or paranasal sinus involvement with seven cases exhibiting maxillary and orbital involvement also. Clinical presentation and risk factors.

Duration of clinical symptoms ranged from two days to three months with late presentation seen among patients having ocular involvement.

Nasal stuffiness, blockage, a nasal mass, and discharge were the common clinical presentations of our patients. In addition to this, patients having orbital involvement (seven cases) showed periorbital pain, swelling, congestion, watering of the eye, and restricted ocular movement along with a diminution of vision. Fundus examination in these cases revealed features of optic disc edema and central retinal artery occlusion (CRAO) in four cases.

Relevant history for co-morbid conditions was seen in 55.5% (25/45 cases) of the patients. Most of whom (88%; 22/25) were on anti-hyperglycemic treatment. Six of these patients in addition were on antihypertensives also.

Laboratory investigations

COVID-19 testing report could be retrieved only from 27 cases from the record file, of which 15 cases (33.33 %) showed positive results on RT-PCR (Reverse transcriptase- polymerase chain reaction) testing. Five cases in 2020 and seven cases till June 2021 tested negative on admission. Repeat testing was not done in any of these cases.

Hematological and relevant biochemical parameters were available in just a few of the patients (Table 1), most of them were the ones who underwent ophthalmic exenteration.

**Table 1:** Summarises the different investigations and interpretations in the study group. CRP: c-reactive protein

Tests (Normal value)	Test done (n/45)	Abnormal Values
Complete Blood Count	33	6 (Anemia and neutrophilic leucocytosis)
D-dimer (220 - 500 ng/ml)	8	5 (Raised 500-1120 ng/ml)
CRP ( < 10 mg/l )	15	14 ( Raised 30.1 to 277 mg/l)
Renal Function Tests (RFT)- blood urea (6- 24 mg/dl)	31	24 (Raised 26-130 mg/dl)
RFT- S creatinine (0.6 - 1.3 mg/dl)	31	24 (Raised 1.42 - 2.3 mg/dl)
Random Blood Sugar (< 140 mg/dl )	22	22 (Raised 180-250 mg/dl)
HbA1c ( < 5.7% )	4	4(Raised 9.9 to 13.3 gm/dl)

The majority of the laboratory parameters were deranged in patients who underwent ophthalmic exenteration. The details have been mentioned in Table 2.

**Table 2:** Depicting demographic, hematological, and biochemical parameters in ophthalmic exenteration patients CVA: cerebro vascular accident; CRP: c-reactive protein

	1	2	3	4	5	6	7
Age (yr)	45	50	54	55	55	56	62
Gender	M	M	F	M	M	M	M
Comorbidity	T2DM, HT, CAD	T2 DM	HT, DM (REC)	DM, HT	NA	T2DM, CVA	T2DM
Hb g/dl	9.6	13.9	8.2	6.6	NA	10.2	9.5
Total Leucocyte Count / mm^3^	12.6	15.3	8.2	10.5	NA	8	5.8
Differential Leucocyte Count %	P80L15	P70L23	P64L30	P80L20	NA	P79L12	P63L27
Platelet count/ mm^3^	N	N	N	N	NA	N	N
Blood urea mg/dl	65	26	40	73	NA	59	130
S. creatinine mg/dl	1.92	1.42	1.51	1.88	NA	1.54	2.3
HbA1c gm/dl	13.1	12.4	>13	NA	NA	NA	9.9
CRP mg/l	240	178	131.6	30.1	NA	NA	277.9
D-dimer ng/ml	1120	1087	1202	898	NA	600	632
Na meq/l	142	133	142	133	NA	134	126
K meq/l	3.1	4.4	3.1	3.2	NA	3.6	3.1

Radiological findings could be retrieved in 5/7 patients who underwent ophthalmic exenteration. The findings were almost common to all and showed involvement of nasal and orbit-o-maxillary areas with a soft swelling in intra-orbital tissues, proptosis, and necrosis to various extents. Secondarily, brain involvement occurred in 3/7 cases who also developed cerebro vascular accident (CVA). Cavernous sinus thrombosis (CVT) occurred in 1/7 of patients.

**Figure 1:**
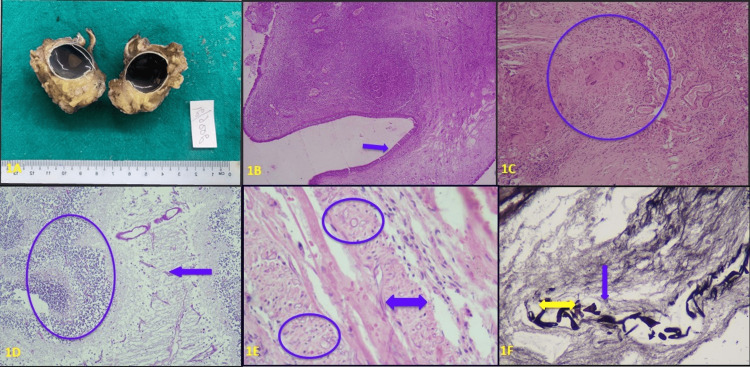
Shows images depicting the histomorphology of mucormycosis 1A- Shows bisected specimen of ophthalmic exenteration, 1B- Photomicrograph showing nasal cavity mucosa (blue arrow) revealing moderately dense chronic inflammatory infiltrate in the stroma (x 20; H&E stain), 1C- Photomicrograph showing granulomatous reaction with giant cells (blue circle) within the nasal mucosal parenchyma (x 40; H&E stain), 1D- Photomicrograph showing infiltration of retinal tissue [blue circle] by broad-based stout hyphae morphologically resembling mucormycosis [blue arrow] (x 40; PAS stain), 1E: Photomicrograph showing evidence of vascular invasion [blue double-headed arrow: vessel wall] of the nasal mucosa by hyphae morphologically resembling mucormycosis [blue circle] (x 40; H&E stain), 1F- Photomicrograph showing the presence of broad-based stout hyphae morphologically resembling mucormycosis (Double headed yellow arrow) in the vessel wall [blue arrow] (x 40; GMS stain)

Gross and microscopic findings were noted from the department records in all 45 cases and the slides retrieved were reviewed by the authors. Gross specimens were mostly either excision biopsies or functional endoscopic sinus surgery (FESS) curetting. Ophthalmic exenteration specimens were received in seven cases (Figure 1A). Maxillectomy was also performed in an orbital exenteration case. Diagnosis of mucormycosis along with mixed fungal infection was done mainly based on histopathological evidence. KOH preparation and culture were done in a few cases only, which further confirmed the diagnosis of fungal pathology. Direct microscopy, serology, immunohistochemistry, breath test, or molecular testing was not done in any of the cases. Microscopic examination in almost all the cases showed extensive tissue necrosis admixed with acute and chronic inflammatory infiltrate along with evidence of bone destruction. Granulomatous reaction with giant cells, in addition, was seen in 6 (13.9%) cases (Figures 1B, 1C). All of the 45 histopathology specimens from rhino-orbital tissues in our study showed fungal hyphae morphologically resembling Mucorales genera as non-pigmented, broad, thin-walled, ribbon-like, predominantly aseptate hyphae with right-angle branching in PAS (Figure 1D). Thirty-one (31/45) of the tissue biopsies solely exhibited the presence of mucormycosis. Angioinvasion by the mucor species was seen in slightly more than half of the cases (52.08%) (Figures 1E, 1F).

**Figure 2:**
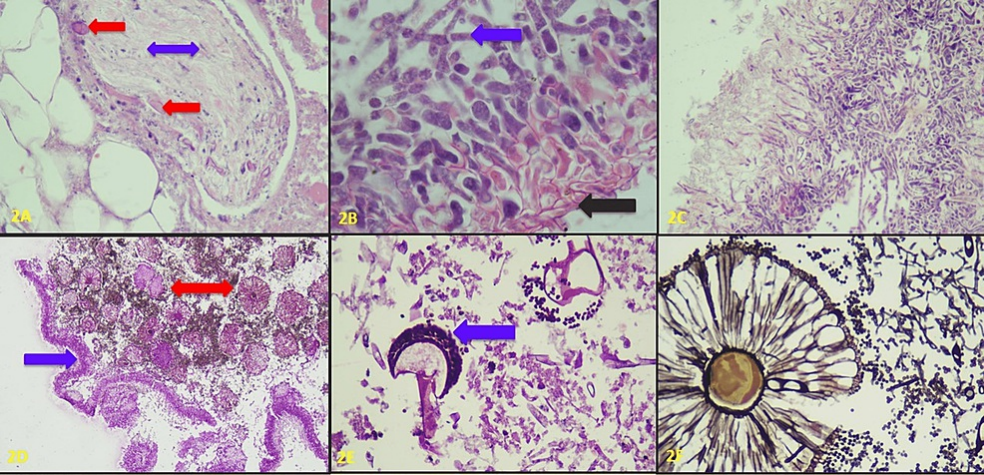
Depicts histomorphology of various fungi 2A- Photomicrograph showing optic nerve [double headed blue arrow] involvement by mucormycosis [red arrows] (x 40; PAS stain), 2B- Photomicrograph showing mixed fungal infection morphologically resembling mucormycosis [black arrow] and aspergillus [blue arrow] (x 40; PAS stain), 2C- Photomicrograph showing slender fugal hyphae, some with acute angle branching morphologically resembling aspergillosis (x10; H&E), 2D- Photomicrograph showing nasal mucosal lining [blue arrow] with underlying tissue showing presence of fruiting bodies [double headed red arrow; (x 20; PAS stain)], 2E & F: Photomicrograph 2E [blue arrow; PAS stain; x 20] and 2F (GMS stain; x 40) showing conidiophores of aspergillus species.

The optic nerve was involved in the infective process and showed the presence of mucormycosis in six of the seven (85.1%) ophthalmic exenteration specimens (Figure 2A). Histopathological evidence of fungal hyphae characteristic of mucor species was seen in 31 cases, mucor with aspergillus species in nine cases (Figure 2B), mucor with candida species in two cases, and mucor with aspergillus and candida in three cases. Records of KOH mount and culture could only be retrieved in 14 cases, 12 of which confirmed fungal infection. There were seven cases of mucormycosis (all ophthalmic exenteration cases along with four cases showing candida morphology and in one case aspergillus fungus was grown. Aspergillus revealed slender hyphae with acute angle branching, fruiting bodies, and conidiospores in H&E stain and PAS as well as on GMS stain (Figures 2C, 2D, 2E, 2F). Candida was seen as pseudo-hyphae and yeast forms.

The patients in this study were started on intravenous anti-fungal treatment (amphotericin B) and were discharged in satisfactory condition.

## Discussion

COVID-19 has emerged as a global pandemic. Extensive and injudicious use of antibiotics, steroids, and comorbidities has developed a more worrisome feature of this pandemic in form of superimposed fungal infections, especially during the second wave [3, 5]. The most common fungal pathogens affecting humans are reported to be Aspergillus, and Candida followed by Mucor, Cryptococcus, and Pneumocystosis (PjP) species [4]. These pathogens normally reside in the manure, starchy foods, soil, and fruits and commonly colonize the oro-naso-pharyngeal mucosa and para-nasal sinuses (PNS) of asymptomatic patients and only cause disease, whenever there is a defect in the immunity in any form [10, 19-20]. Involvement of the rhino-orbital area and lung by these organisms has resulted in increased morbidity and mortality in COVID-19-affected patients with a significant upsurge of infection especially by mucor species [5-10]. Aspergillus and candida were the commonest species reported during the first wave, however, a marked upsurge of rhino-orbital mucormycosis (ROM) during the second wave of COVID-19 was attributed to immunosuppression by the virus-causing hypoxic environment, with high glucose and ferritin level and defective phagocytic activity of leukocytes by the new variant of SARS-CoV-2 [4-5, 9, 11, 21-22]. The present study also showed an increase in the incidence of COVID-19-associated mucormycosis during the second wave as compared to the incidence during the pre-COVID era and first wave (from 13.3% to 31.1% to 68.8%). A thorough literature search showed only a single study by Nucci et al., revealing a similar increase in fungal infections among COVID-19-infected patients in the pre-pandemic era [23].

The present study was done based on the observation regarding the remarkable increase in rhino-orbital mucormycosis in routine biopsies during the COVID-19 pandemic as compared to the previous year's findings (six cases only in 2019). Even though many studies have been published since the pandemic, most of them are either reported as case studies/series or as literature reviews. However, there aren’t many regarding the spectrum of fungal infections in a rural tertiary care center set up in North India, hence, an effort was made to compile the data depicting the same. 

The demographic profile showed a majority of the patients in our study to be males with their ages ranging from 23-70 years which was in accordance with various other studies [9-13].

All the patients in our study gave a brief history of mild to moderate symptoms pertaining to COVID-19 infection. Non-specific symptoms like nasal stuffiness, blockage, facial swelling, a nasal mass, and discharge were seen in most of the patients as observed in other studies [6, 8, 10]. Patients with orbital involvement showed a diminution of vision, increased watering of eyes, and restricted ocular movements.

Orbital infections are extremely rare and usually develop from the infected adjacent paranasal sinuses or through direct traumatic inoculation into the orbit [6, 15, 24-25]. Apart from the usual symptoms, sudden blindness can occur due to central retinal artery occlusion, thrombosis of posterior ciliary arteries, infarction of the intra-orbital part of the optic nerve, or direct fungal invasion of the intracranial part of the optic nerve or optic chiasma [23, 26]. There were seven cases of orbital mucormycosis requiring orbital exenteration in our study, all of whom showed partial to complete loss of vision in the affected eye. There were four cases that showed features of optic disc edema and central retinal artery occlusion (CRAO) as mentioned in the literature. Secondarily brain involvement was seen in three cases and cavernous sinus thrombosis in one patient. Similar findings were also reported in other studies [24-26].

The time duration between the occurrence of COVID-19-related manifestations and the development of rhino-orbital fungal infection in various studies ranged from three to 42 days [1, 7, 15, 17]. Which was comparable to the present study (two days to three months).

Details regarding COVID-19 testing were documented in only 27 cases of which only 15 (33.33 %) showed positive results on reverse transcription-polymerase chain reaction (RT-PCR) testing. This was discordant with a majority of the studies, whereby almost all the patients with associated mucormycosis showed COVID-19 positivity [1-3, 7, 9]. Negative testing in 12 (12/27 cases) of the cases could be attributed to their delayed presentation to the out patient department (OPD). Non-documentation of the report could be the reason for the rest of the cases (18 cases).

The majority of the previous and recent studies in the literature reflected immune-compromised status or comorbid conditions like diabetes mellitus (DM), chronic hypertension, and kidney diseases to be predominantly responsible for the development of fungal diseases [9-10, 13,-15, 17]. Slightly more than half (51.3%) of the patients in the present study did show associated co-morbidities with DM taking precedence which was similar to other studies [9, 17, 22, 24]. The increased association of DM was accredited to limited access to routine diabetes care because of lockdown resulting in cytokine storm causing beta cell injury and hence leading to poor glycemic control [27].

Among the other cofactors contributing to rhino-orbital fungal infections, steroid intake and injudicious use of antibiotics have been mentioned in various studies [5-8]. No data regarding misuse of antibiotics was documented while going through the patient's record, however, a history of treatment with steroids was seen among five patients (5/45) who underwent ophthalmic exenteration.

Contaminated oxygen was considered a source causing fungal infections. However, none of our patients required mechanical ventilation or any kind of oxygen support which was also supported by a few studies which also showed the same [9, 17, 22]*.*

Similar to a few studies, no history of any comorbid condition was seen in the rest of the 18 patients in our study. This diverts our attention to other unknown factors which can be responsible for fungal co-infections [14, 16].

Deranged hematological and biochemical laboratory findings in the form of anemia, neutrophilia, lymphopenia, raised postprandial blood sugar (PPBS), HbA1c, serum creatinine, c-reactive protein (CRP), pro-calcitonin, D-Dimer, and IL-6 has been associated with COVID-19 disease [10, 17, 19]*.* Medical records regarding the same were available in only a few cases, the majority of which showed deranged parameters, which were in concordance with the above-mentioned studies. The reason for the non-availability of the laboratory findings could be attributed to a few reasons according to the authors, the most common being non-documentation of the laboratory results due to extensive workload. The other reason could be, that the rhino-nasal biopsy was more of an out-patient procedure with patients not requiring stringent pre-operative investigations whereas patients who had ophthalmic involvement required major surgery and hence the detailed investigations.

CT scan of PNS was done in five ophthalmic exenteration cases only. It showed nasal and orbito-maxillary involvement with swelling in intra-orbital tissues, along with necrosis which was compatible with other studies [9, 28].

Microscopic examination of both FESS and ophthalmic exenteration showed infiltration by mucormycosis species in a majority (31/45) of the cases. Similar findings were seen in most of the studies [8, 12-13, 15]. A few cases in addition showed infiltration by mixed fungal profiles comprising Candida, and Aspergillus similar to other authors [1, 7-10, 13, 17].

Rhino-orbital mucormycosis (ROM) can be non-invasive (mycetoma) or invasive with the latter exhibiting acute and chronic (granulomatous and non-granulomatous) forms. More than half of our cases (55.5%) were the invasive type with granulomatous pathology seen in six cases. The present study showed a variable amount of tissue necrosis (66.6%) on histopathology which contained the maximum density of fungal organisms. Similar findings were also observed in other studies [8, 12, 20, 25]. Optic nerve involvement was observed in six out of seven cases of exenteration specimens in the present study in agreement with other studies [9, 10, 12, 14].

Tissue for KOH mount and fungal culture could be retrieved in only 14 cases, seven of which confirmed the histological diagnosis of mucormycosis. The negative yield on KOH mount/ culture is assumed to be due to the aggressive processing of the specimen before the plating medium. Similar findings have also been documented by a few other authors [25, 29-30].

Limitations

This was more of an observational single-center study in a rural setup with a smaller number of cases. Relevant and mandatory clinical and laboratory data required for the study were also missing in some of the patient's medical records. From the author's point of view, the probable reason for missing data could be increased workload which might have led to the non-entry of the relevant data in the medical record files.

## Conclusions

Upsurge in rhino-orbital fungal infections has been seen among COVID-19 patients and is commonly attributed to immune dysregulation. Injudicious use of antibiotics/ steroids and with no treatment protocol in place due to the virus being novel, along with associated co morbid conditions (DM and HT) added to crisis. Histopathology plays a very important role with regards to aiding in early diagnosis and hence prompt and appropriate treatment of the fungal infections as seen in our study.

An interesting thing which was also noted in our study was that about one third of the patients did not have decreased immunity which attracts our attention to think and search about other parameters which can be the causative factors for these co-infections in Indian scenario. A large-scale multi-centric study would help to gain useful data on this deadly disease so as to combat secondary infections.
